# *Scutellaria baicalensis* Extracts and Flavonoids Protect Rat L6 Cells from Antimycin A-Induced Mitochondrial Dysfunction

**DOI:** 10.1155/2012/517965

**Published:** 2012-08-30

**Authors:** A-Rang Im, Young-Hwa Kim, Md. Romij Uddin, Hye Won Lee, Seong Wook Chae, Yun Hee Kim, Woo Suk Jung, Bong Ju Kang, Chun Sun Mun, Mi-Young Lee

**Affiliations:** ^1^Korea Institute of Oriental Medicine, 461-24 Jeonmin-dong, Yuseong-gu, Daejeon 305-811, Republic of Korea; ^2^Department of Crop Science, Chungnam National University, Daejeon 305-764, Republic of Korea; ^3^School of Chinese Materia Medica, Beijing University of Chinese Medicine, Beijing 100102, China

## Abstract

Antimycin A (AMA) damages mitochondria by inhibiting mitochondrial electron transport and can produce reactive oxygen species (ROS). ROS formation, aging, and reduction of mitochondrial biogenesis contribute to mitochondrial dysfunction. The present study sought to investigate extracts of *Scutellaria baicalensis* and its flavonoids (baicalin, baicalein, and wogonin), whether they could protect mitochondria against oxidative damage. The viability of L6 cells treated with AMA increased in the presence of flavonoids and extracts of *S. baicalensis*. ATP production decreased in the AMA treated group, but increased by 50% in cells treated with flavonoids (except wogonin) and extracts of *S. baicalensis* compared to AMA-treated group. AMA treatment caused a significant reduction (depolarized) in mitochondrial membrane potential (MMP), whereas flavonoid treatment induced a significant increase in MMP. Mitochondrial superoxide levels increased in AMA treated cells, whereas its levels decreased when cells were treated with flavonoids or extracts of *S. baicalensis*. L6 cells treated with flavonoids and extracts of *S. baicalensis* increased their levels of protein expression compared with AMA-treated cells, especially water extracts performed the highest levels of protein expression. These results suggest that the *S. baicalensis* extracts and flavonoids protect against AMA-induced mitochondrial dysfunction by increasing ATP production, upregulating MMP, and enhancing mitochondrial function.

## 1. Introduction

The generation of ROS, which are products of respiration, is believed to contribute substantially to aging [1, 2]. Oxidative stress and mitochondrial dysfunction are important factors that contribute to aging [3]. A series of protein complexes (I–IV) is embedded in the inner mitochondrial membrane generating a proton gradient known as the mitochondrial membrane potential (MMP) [4]. The mitochondrial respiratory chain is a major site of ROS production in the cell. Generations of ROS play an important role in mitochondrial dysfunction and represent putative targets of antiaging strategies [5]. Antimycin A (AMA) damages mitochondria in many cell types by inhibiting mitochondrial electron transport [6, 7]. Complexes I and III of the mitochondrial electron transport chain are the major sites for ROS production. In mitochondria, AMA binds to the Qi site of cytochrome c reductase, thereby the oxidation of ubiquinol in the electron transport chain of oxidative phosphorylation. AMA is known to cause the leakage of superoxide radicals from rat liver mitochondria [8].

The dried root of *S. baicalensis* is rich in flavonoids, containing over 30 different kinds of flavonoids. Phytochemical investigations revealed that flavonoids primarily comprise baicalin, baicalein, and wogonin. The antioxidants and anti-inflammatory flavonoids, baicalin, baicalein, and wogonin are present in abundance in this medicinal herb [9–13]. Ethanol extracts of *S. baicalensis* prevent oxidative damage and neuroinflammation, and aqueous extracts have oxidative effects in cultured human umbilical vein endothelial cells [14, 15]. Baicalein, baicalin, and wogonin each affect mitochondrial function [16]. Baicalein protects mitochondria against oxidative damage via induction of manganese superoxide dismutase and hydrogen peroxide-induced oxidative stress in HS-SY5Y cells [17, 18]. Baicalin also acts as a prooxidant and induces caspase-3 activation and apoptosis via the mitochondrial pathway [19]. Despite the many studies of *S. baicalensis* extracts and flavonoids isolated from them, little is known about their effects on antimycin A-induced mitochondrial dysfunction. The goal of the present study was to increase our understanding of how and how much of these compounds mitigate the mitochondrial damaging activity of AMA towards mitochondria by assessing the protective effect from both the extracts and flavonoids (baicalein, baicalin, and wogonin) of *Scutellaria baicalensis* against AMA-induced L6 cells.

## 2. Materials and Methods

### 2.1. Materials

Baicalein, baicalin, and wogonin were purchased from Wako Pure (Tokyo, Japan). AMA and DAPI solutions were purchased from Sigma Chemical (St. Louis, MO, USA). Dulbecco's modified Eagle's medium (DMEM) and fetal bovine serum (FBS) were purchased from Gibco BRL (Grand Island, NY). CellTiter Aqueous One Solution Cell proliferation assay kit ([3-(4,5-dimethylthiazol-2-yl)-5-(3-carboxymethoxyphenyl)-2-(4-sulfophenyl)-2H-tetrazolium, inner salt; MTS) was purchased from Promega Co. (Madison, WI, USA). ATP levels and MMP were determined using a Luminescence ATP detection kit (PerkinElmer, Waltham, MA, USA) and the JC-1 mitochondrial membrane potential detection kit (Biotium, Hayward, CA, USA). Mitotracker and MitoSOX were purchased from Invitrogen Molecular Probes (San Diego, CA, USA). The pAMPK primary antibody and a secondary antibody (anti-goat and rabbit) were purchased from Santa Cruz Biotechnology Inc. (Santa Cruz, CA, USA). The PGC1*α* and SIRT1 antibodies were obtained from Abcam Ltd (Cambridge, UK).

### 2.2. Preparation of *S. baicalensis* Extracts

Aqueous extracts of *S. baicalensis* were prepared by sonicating the dried ground powder (150.58 g) that was suspended in distilled water for 2 h. The process was repeated three times. The suspension was lyophilized, yielding 78.45 g of water extract. A 70% ethanol extract was prepared by sonicating the dried ground powder (150.01 g) suspended in 70% ethanol solvent (v/v % in water), and the suspension was processed as described for the aqueous extract yielding 35.28 g.

### 2.3. Cell Culture

The L6 skeletal muscle cell line was purchased from the Korean Cell Line Bank (Seoul, Korea) and maintained at subconfluence at 37°C in a humidified atmosphere of 95% air and 5%  CO_2_. The cells were grown in DMEM with 10% FBS containing 100 units/mL of penicillin and 100 *μ*g/mL of streptomycin.

### 2.4. Cell Viability and MTS Assay

L6 cells were plated at a density of 1 × 10^4^ cells/well in DMEM containing 10% FBS in a 96-well plate and were incubated at 37°C for 24 h. Cells were treated with varying concentrations of *S. baicalensis* extracts in a 96-well plate after additional incubation at 37°C. Cell viability was determined after 24 h by reduction of MTS to its formazan product. After removing the medium, 200 *μ*L DMEM containing MTS was added to each well, and samples were then incubated at 37°C for 60 min. The absorbance of the reaction at 490 nm was determined using a microplate fluorometer (Molecular Devices, Sunnyvale, CA, USA). To determine whether samples could protect cells against AMA, varying concentrations of extracts were added to the plates 1 h after adding 100 *μ*g/mL AMA. The MTS assay was performed 24 h later as described previously.

### 2.5. ATP Assays

Varying amounts of extracts were added to cells in a 96-well white plate for 1 h before adding 100 *μ*g/mL AMA. Total cellular ATP content was determined using an ATP luminescence detection kit and a luminometer [20]. The values, compared with an internal standard, are expressed as percentages of untreated cells (control).

### 2.6. Mitochondrial Membrane Potential

To detect changes in MMP, JC-1 was used as an indicator of mitochondrial function. The dye 5, 5′, 6, 6′-tetrachloro-1, 1′, 3, 3′ tetraethylbenzimidazolylcarbocyanine iodide (JC-1) fluoresces red or green, respectively, when it aggregates in healthy mitochondria with high membrane potentials or exists as a monomer in mitochondria with diminished membrane potential. Cells were seeded in 96-well plates at 1 × 10^4^ cells/well. MMR was measured using a JC-1 mitochondrial membrane potential detection kit [21]. Before adding JC-1 the medium was aspirated from the plates and adherent cells washed with PBS. The plates were incubated at 37°C for 20 min after the addition of 100 *μ*L of 1× JC-1 reagent into the wells. Cells were washed twice with PBS, and then PBS was added in an amount sufficient to cover the cell layer. Red fluorescence (excitation, 550 nm, and emission, 600 nm) and green fluorescence (excitation, 485 nm, and emission, 535 nm) were determined using a Softmax Pro fluorescence plate reader (Molecular Devices, Sunnyvale, CA, USA). The ratio of red-to-green fluorescence in dead cells and in cells undergoing apoptosis is decreased compared with healthy cells. For confocal microscopy, 1× of JC-1 was added to treated cells for 15 min at 37°C. Cells were imaged using Olympus FV10i-LIV confocal microscopes (Olympus, Tokyo, Japan). In live nonapoptotic cells, mitochondria appeared red due to aggregation of the JC-1 reagent. The red aggregates are excited at 559 nm and emit at 570–620 nm. In apoptotic and dead cells, the dye is monomeric and emits at 490–540 nm when excited at 473 nm.

### 2.7. Mitochondrial Superoxide (MitoSOX)

We used Mitotracker Red reagent for determining mitochondrial superoxide levels. Cells were plated at 1 × 10^4^ cells on white plates for quantitating fluorescence, and 1 × 10^3^ cells were added to cover slips for confocal analysis. The medium was removed, and cells were washed with PBS before measurements. Cells were incubated with 5 *μ*M MitoSOX Red for 20 min at 37°C. MitoSOX Red has excitation/emission maxima of approximately 510/580 nm.

### 2.8. Confocal Microscopy Analysis

Cells were plated at 1 × 10^3^ cells on cover slips. The medium was removed, and cells were washed with PBS first. Cells were incubated for 45 min with 10 *μ*M Mitotracker Red and 1 *μ*g/mL DAPI for detecting mitochondria. Cells were visualized by emission at 598 nm (excitation at 578 nm). DAPI fluorescence was determined at an exciting wavelength of 359 nm, and emission was detected at 461 nm.

### 2.9. PGC-1*α*, SIRT1, and pAMPK Expression

Cells were incubated with varying concentrations of extracts and 100 *μ*g/mL of AMA harvested and lysed in RIPA buffer (T&I, Seoul, Korea). Protein concentrations were determined using the Protein Assay Reagent (Bio-Rad, Hercules, CA, USA). Proteins were separated by SDS-PAGE and transferred to PVDF membranes. The membrane was incubated with the primary antibody (1 : 1000) overnight and the secondary antibody (1 : 5000) for 2 h. The blot was then developed using Luminol Enhancer solution (GE Healthcare, Waukesha, USA), visualized using an ImageQuant LAS 4000 mini (GE Healthcare, Waukesha, USA), and quantified by using Image J densitometry software (Rasband, W.S., ImageJ, U. S. National Institutes of Health, Bethesda, Maryland, USA, http://imagej.nih.gov/ij/, 1997–2011).

### 2.10. Statistical Analyses

All data are expressed as mean ± standard deviation of at least three independent experiments. Statistical analysis was performed using SPSS 20.0 (SPSS, Chicago, IL) by using one-way ANOVA followed by Tukey's post hoc test. Data were considered statistically significant at *P* < 0.05.

## 3. Results

### 3.1. *S. baicalensis* Extracts and Flavonoids Protect against Antimycin A Toxicity

**Figure 1:**
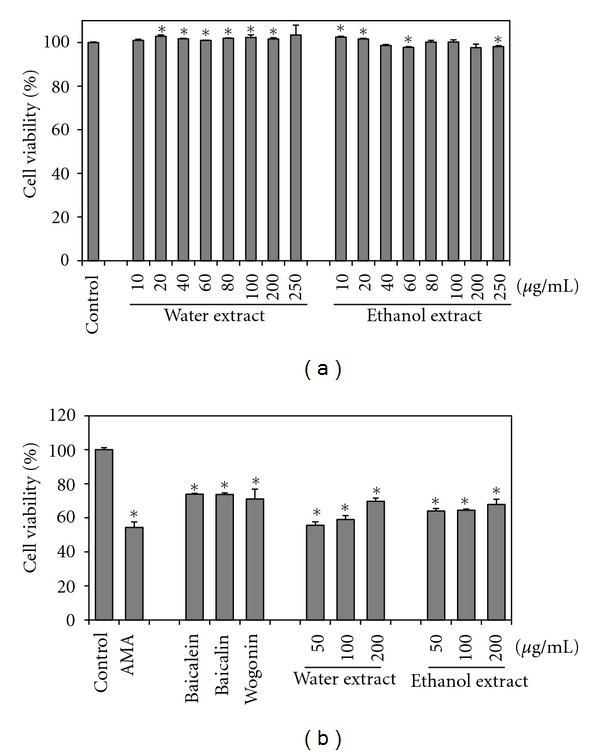
Effects of *S. baicalensis* extracts on cell viability and protective effects of flavonoids on L6 cells treated with AMA. (a) Cell viability of *S. baicalensis* extracts. (b) Cell-protective effect of samples treated with AMA. Cells were pretreated with baicalein, baicalin, and wogonin (50 *μ*g/mL each) for 1 h and pretreated with extracts at 50, 100, 200 *μ*g/mL each for 1 h, and then 100 *μ*g/mL of AMA was added.

Cell viabilities were determined on L6 cells by MTS assay using water and 70% ethanol extracts from *S. baicalensis*. No cytotoxicity was observed in the cell from the extracts of *S. baicalensis* (Figure 1(a)). AMA toxicity significantly reflects the drug's damaging effects on mitochondria. We then determined the dose and time of exposure to 100 *μ*g/mL AMA required to reduce cell viability by 50% after 24 h incubation (data not shown). Cells were then treated with 100 *μ*g/mL AMA in the presence of *S. baicalensis* extracts or individual flavonoids. Baicalein, baicalin, and wogonin significantly increased cell viability by 20% compared with AMA alone (Figure 1(b)). Here all flavonoids performed almost the same for cell viability. Cell viability was increased by addition of 50 *μ*g/mL to 200 *μ*g/mL of water and 70% ethanol showing slightly lower compared to flavonoids, and within the extracts treatment, water extracts performed slightly higher for cell viability compared to ethanol extracts treatment.

### 3.2. Effects of *S. baicalensis* Extracts and Flavonoids on ATP, ADP Levels

**Figure 2:**
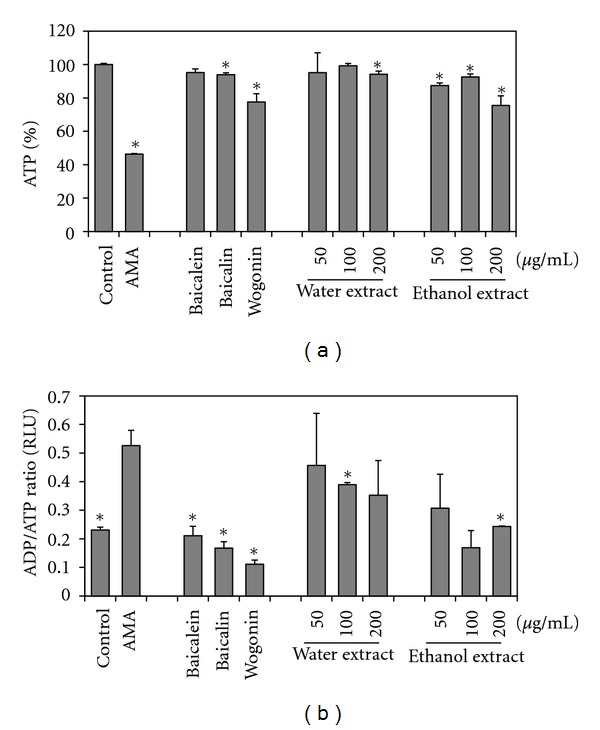
Effects of extracts on total cellular ATP levels and ADP/ATP ratio. (a) Total ATP levels in L6 cells treated with AMA. Cells were pretreated with baicalein, baicalin, or wogonin (each at 50 *μ*g/mL) for 1 h. Cells were pretreated with extracts (50, 100, 200 *μ*g/mL each) for 1 h, and then 100 *μ*g/mL of AMA was added. ATP levels were measured using the ATP Lite luminescence-based assay in which results are reported as a percentage of the control. (b) ADP/ATP ratio in L6 cells was determined using the EnzyLight ADP/ATP ratio assay kit. Data were expressed as a percentage of control. *P < 0.05.

To determine whether the extracts or flavonoids affected energy production, we measured ATP levels. ATP production decreased in the AMA-treated group but increased significantly in cells treated with flavonoids and extracts of *S. baicalensis* (Figure 2(a)). ATP production increased by 50% in cells treated with flavonoids (except wogonin) and extracts of *S. baicalensis* compared to AMA-treated group (Figure 2(a)). Among the treatments water extract at 100 *μ*g/mL yielded the highest ATP production (Figure 2(a)). The ADP/ATP ratio was substantially elevated in AMA-treated cells compared with controls (Figure 2(b)). The three flavonoids decreased the ADP/ATP ratios, by wogonin in particular. The ethanol extracts reduced this ratio to a great extent.

### 3.3. Mitochondrial ROS Production

**Figure 3:**
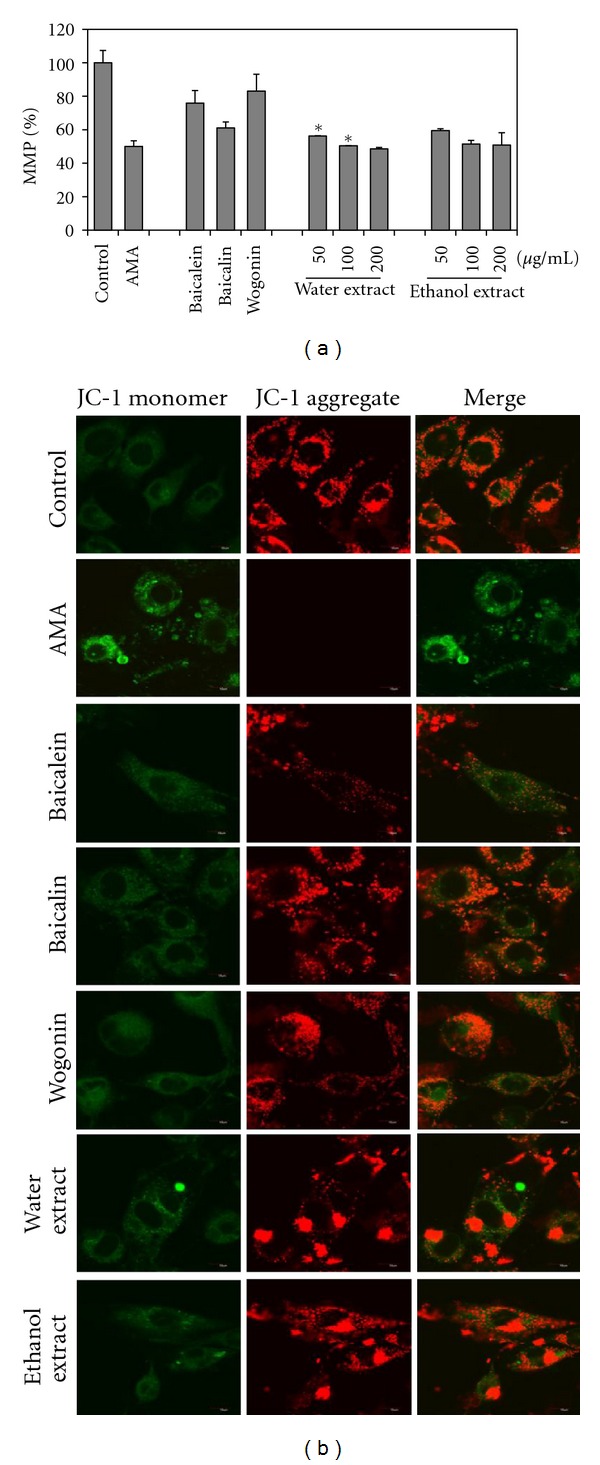
*S. baicalensis* extracts prevented polarized MMP. Cells were treated with AMA alone for 24 h. (a) Cells were pretreated with baicalein, baicalin, wogonin (50 *μ*g/mL each) for 1 h and with extracts (50, 100, 200 *μ*g/mL each for 1 h), and then 100 *μ*g/mL of AMA was added. Red fluorescence (excitation 550 nm, emission 600 nm) and green fluorescence (excitation 485 nm, emission 535 nm) were determined by using a fluorescence plate reader. (b) Confocal images show JC-1 fluorescence (60 × 2.5). Cells were pretreated with 50 *μ*g/mL for 1, and then 100 *μ*g/mL of AMA was added. Depolarized mitochondria were detected by green fluorescence, and polarized mitochondria were detected by red fluorescence. Data are expressed as a percentage of the control. *P < 0.05.

Disruption of MMP results from mitochondria dysfunction and induction of apoptosis. AMA depolarizes mitochondrial membrane. To study the direct effect of *S. baicalensis* extracts on AMA-induced oxidative stress, we determined MMP. Cells were incubated with flavonoids and extracts of *S. baicalensis* for 1 h, and then 100 *μ*g/mL of AMA was added and cells incubated for 24 h. The red AMA signal decreased greatly, and the red/green signal ratio also decreased using JC-1 (Figure 3(a)). Cells in control treatment have both green and red signal, showing mitochondria exactly (Figure 3(b)). AMA treatment for 24 h caused a significant reduction in JC-1 ratio indicating a depolarized MMP, whereas flavonoids and extracts treatment induced a significant increase in MMP as follows: by 83.1%, 75.9%, 61.1%, 59.5%, and 56.3% using wogonin, baicalein, baicalin, ethanol extracts, and water extracts, respectively (Figure 3(a)) as indicated by an increase in red JC-1 fluorescence (Figure 3(b)).

### 3.4. Changes of Mitochondrial Superoxide Levels

**Figure 4:**
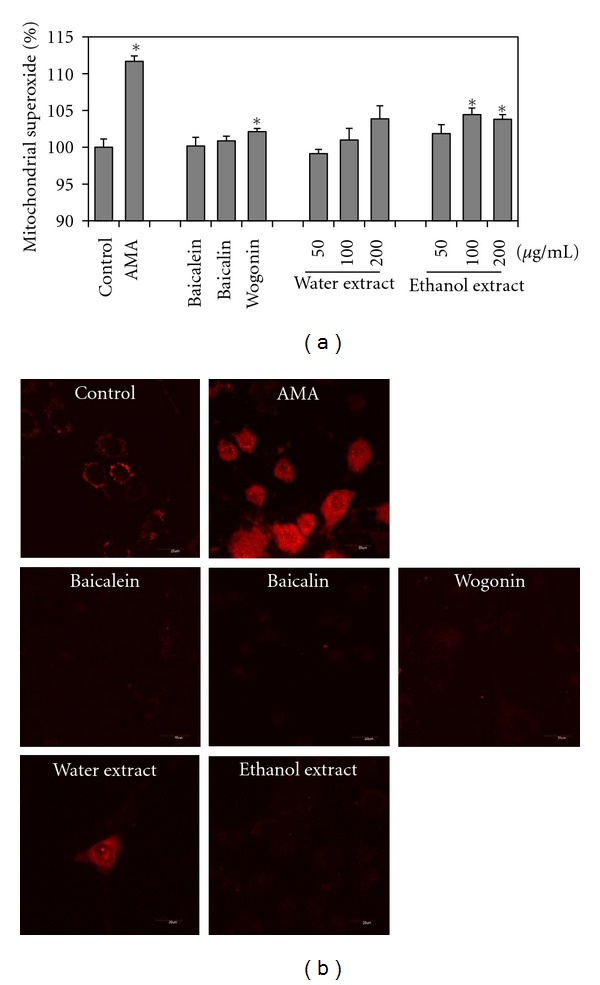
Determination of mitochondrial superoxide production measured using MitoSOX Red. (a) Cells were pretreated with baicalein, baicalin, wogonin (50 *μ*g/mL each) for 1 h or with extracts (50, 100, 200 *μ*g/mL each) for 1 h, and then 100 *μ*g/mL AMA was added. (b) Determination of mitochondrial superoxide production measured by confocal microscopy (60 × 1.5). Samples were pretreated with 50 *μ*g/mL for 1 h, and then 100 *μ*g/mL of AMA was added. Data were expressed as a percentage of control. *P < 0.05.

 Quantitative measurements of the mean intensity from the AMA-induced cells demonstrated a 111.7% increase compared with control cells (Figure 4(a)). In contrast, mitochondrial superoxide levels decreased around 10% when cells were treated with flavonoids and extracts. Confocal microscope imaging demonstrated an increase in mitochondrial MitoSOX fluorescence in cells treated with AMA for 24 h (Figure 4(b)). The mitochondria of AMA-induced cells exhibited red fluorescence, indicating the presence of superoxide, whereas the control, flavonoids, and extracts of *S. baicalensis*-treated cells did not show or showed slight red fluorescence indicating a low level of superoxide.

### 3.5. Confocal Analysis of Mitochondria

**Figure 5:**
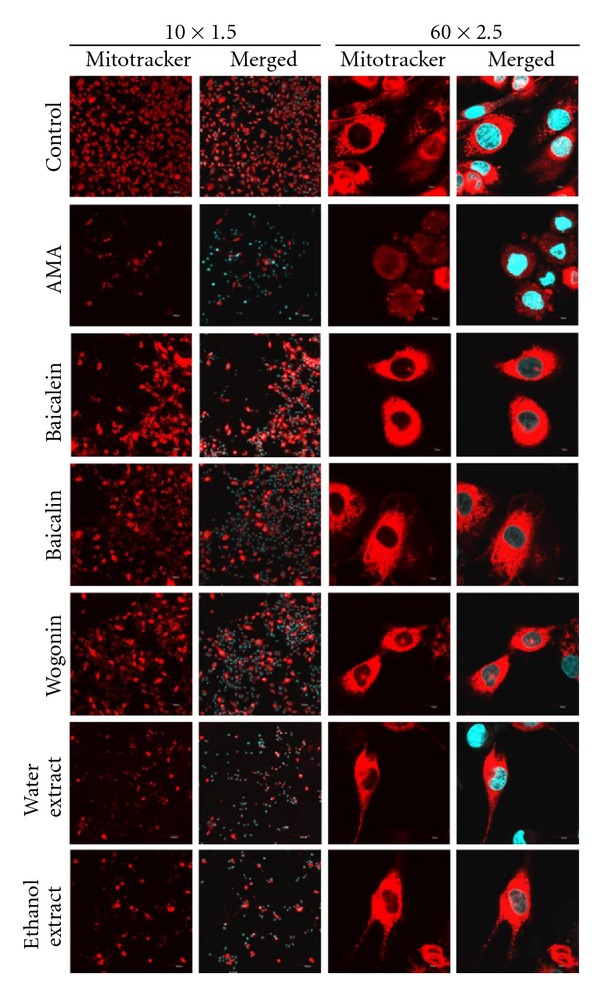
Confocal microscopy of mitochondria. Distribution of Mitotracker red fluorescence indicated mitochondria, and merged images indicated colocalization with DAPI blue fluorescence (10 × 1.5 and 60 × 2.5). Samples were treated with 50 *μ*g/mL for 1 h, and before 100 *μ*g/mL of AMA were added.

 Mitotracker probes can be used to stain the mitochondria of L6 cells with red fluorescence. Confocal observations revealed evenly distributed mitochondrial staining in control cells (Figure 5). AMA-induced cells showed reduced red fluorescence intensity in mitochondria indicating a depolarization of the inner mitochondrial membrane. The mitochondrial staining pattern was restored from that of control cells by 1 h treatment with 50 *μ*g/mL of baicalein, baicalin, and wogonin. Extracts treatment also increased the red fluorescence intensity and membrane permeability to DAPI.

### 3.6. PGC-1*α*, SIRT1, and pAMPK Expression

**Figure 6:**
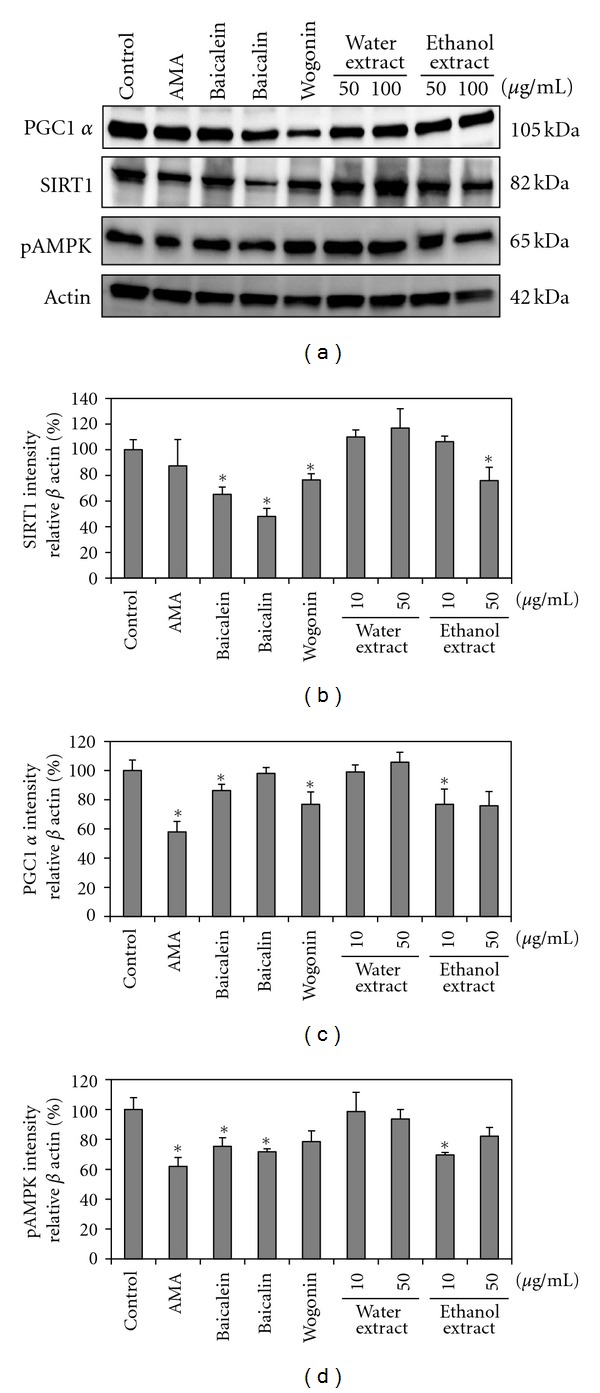
PGC-1*α*, SIRT1, and pAMPK protein expression analysis in L6 cells. Cell lysates were prepared from L6 cells, were pretreated with extracts for 1 h, and then exposed to AMA. The phosphorylated and total protein levels were detected with specific antibodies by Western blotting. *β*-actin served as the loading control. Quantitative analysis was performed by measuring the signal intensity relative to the control (*n* = 3).

PGC-1*α*, SIRT1, and pAMPK were expressed in L6 cells as determined by Western blotting analysis (Figure 6). The expression of each protein was increased by treatment of cells with flavonoids or extracts of *S. baicalensis*-treated cells compared to AMA-treated cells. Among the treatments of flavonoids and extracts, water extracts performed the best for expressing the intensity of protein. PGC-1*α* levels increased when cells were treated with flavonoids and extracts of *S. baicalensis.* In particular, the water extracts and baicalin at 50 *μ*g/mL increased PGC-1*α* by 47.7 and 40%, respectively. SIRT1 intensity increased by 30% and 19% using more water and ethanol extracts of *S. baicalensis* at 50 *μ*g/mL, respectively, compared with AMA cells. All the treated cells with flavonoids and extracts exhibited higher pAMPK intensity compared with AMA cells. Water extract and ethanol extracts at 50 *μ*g/mL increased the intensity of pAMPK by 36.6 and 20.2%, respectively, compared with AMA cells.

## 4. Discussion

 In the present study, we focused on the flavonoids and extracts prepared from *S. baicalensis* for their effect on mitochondrial dysfunction (as indicated by oxidative stress) induced by AMA. Cell viability was increased by the addition of baicalein, baicalin, and wogonin before. These results agree with the findings of others that apocynin protects against AMA-induced cell damage in osteoblastic MC3T3-E1 cells [22]. Mitochondrial dysfunction induced by AMA results in decreased ATP production. Oxidative stress increases respiration and generation of ROS, resulting in ATP depletion [23]. We show here that treatment with baicalein, baicalin, and wogonin before AMA exposure significantly prevented loss of ATP and MMP, suggesting that molecules present in *S. baicalensis* protect cells from mitochondrial dysfunction. Thus, the percentage of extracellular ATP in AMA treated cells was 46.36%, but pretreating samples with flavonoids and extracts of *S. baicalensis* increased this value.

 Mitochondrial ROS production is intimately linked to MMP, such that hyperpolarization increases and promotes ROS production [24]. Our data presented here show that cells treated with *S. baicalensis* extracts exhibited decreased MMP and increased mitochondrial biogenesis. The disruption of MMP in L6 cells is the result of ROS-mediated damage. The MitoSOX Red results indicate that mitochondrial superoxide was increased in AMA-induced cells.

As the major producer and primary target of ROS, mitochondria play an important role in aging. Our present study demonstrates that cells treated with baicalein, baicalin, and wogonin prevented AMA-induced ROS production by mitochondria. Mitotracker Red dyes are being used with increasing frequency for morphological and functional measurements of mitochondria. Here, Mitotracker Red fluorescence delocalized during mitochondrial depolarization and increased following treatment with AMA, indicating a decrease in the capacity of these cells to generate energy-rich reductants. Baicalein, baicalin, and wogonin treatment significantly increased mitochondrial number compared with AMA-induced cells.

PGC-1*α*, SIRT1, and pAMPK have been identified as the major transcription factors controlling expression of mitochondrial genes; however, the signaling pathways between the mitochondria and the nucleus remain to be elucidated [4]. SIRT1 regulates aging and resistance to oxidative stress in the heart *in vivo*, and stimulation of SIRT1 may be considered as an antiaging therapy for the heart [25]. An SIRT1 activator, resveratrol, induces PGC-1*α* activity by facilitating SIRT1-mediated deacetylation [26]. SIRT1 is activated and cooperates with pAMPK to enhance the ability of PGC-1*α* to stimulate mitochondrial biogenesis and function [27]. AMPK directly phosphorylates PGC-1*α*, enhancing its activity at its own promoter and triggering a transcriptional cascade that increases expression of PGC-1*α* and its target genes that are involved in mitochondrial biogenesis [28]. Here, we found that L6 cells treated with flavonoids and extracts of *S. baicalensis* upregulated SIRT1 levels and stimulated pAMPK activity in AMA-induced cells. Activation of AMPK increases the activities of citrate synthase and succinate dehydrogenase, which may regulate mitochondrial biogenesis in response to energy depletion. AMPK phosphorylates PGC-1*α*, a key regulator of mitochondrial biogenesis and metabolism [29]. Quercetin protects against H_2_O_2_-induced cell death and increases mitochondrial biogenesis by upregulating PGC-1*α* and SIRT 1, which regulate mitochondrial activity in human retinal pigment epithelium *in vitro* [30]. Mitochondrial dysfunction, characterized by a decline in cellular ATP, loss of MMP, and decrease of protein content, is central to the execution of cell death.

## 5. Conclusion

In conclusion, the protection of mitochondrial dysfunction by *S. baicalensis* extracts (containing the flavonoids baicalein, baicalin, and wogonin) especially water extracts from AMA-induced oxidative damage and cell death can be attributed to increased ATP levels, regulation of MMP, and increased mitochondrial function mediated by PGC-1*α*, SIRT1, and pAMPK.
